# A Comparative Study of Success Rates of Post and Core Treated Anterior and Posterior Teeth Using Cast Metal Posts

**DOI:** 10.7759/cureus.30735

**Published:** 2022-10-26

**Authors:** Jayant Prakash, Mahesh Suganna Golgeri, Shaista Haleem, Hina Kausher, Prashant Gupta, Pushpraj Singh, Shivakumar G C.

**Affiliations:** 1 Dental Department, Sadar Hospital, Muzaffarpur, IND; 2 Department of Prosthodontics, College of Applied Medical Sciences, Riyadh Elm University, Riyadh, SAU; 3 Department of Applied Medical Sciences, Riyadh Elm University, Riyadh, SAU; 4 Department of Prosthodontics, Dental Lab Technology College of Applied Medical Sciences, Riyadh Elm University, Riyadh, SAU; 5 Department of Oral Medicine and Radiology, Dental Institute – Rajendra Institute of medical Sciences, Ranchi, IND; 6 Department of Dentistry, Government Medical College, Shahdol, IND; 7 Department of Oral Medicine and Radiology, People’s College of Dental Science and Research Centre, Bhopal, IND

**Keywords:** survival rate, restoration, post and core, clinical trial, cast metal post

## Abstract

Background: Post and core restorations are performed (generally after treating the root canals of the tooth) when the majority of the tooth structure has been rendered ineffective to support itself/the crown of the tooth. In this clinical study, we aim to compare the difference between post and core treatment of anterior and posterior teeth and their success/failure rates.

Objective: Our investigation aimed to compare the clinical survival rates of custom-fabricated cast metal post-and-cores in cases of anterior and posterior teeth while controlling for the population's age and gender, type of post material, length of the post, amount of alveolar bone tissue supporting the roots, tooth location in the dental arch, and type of cement used, as well as the effect of opposing dentition and the type of definitive prosthetic treatment received.

Methods: 112 individuals who had received root canal therapy (RCT) and were chosen to receive post and core therapy following their RCT were chosen for the study, and a total of 164 teeth were scheduled to undergo the therapy. All the posts used were made of cast metal, and the patients were divided into two groups: 48 individuals had the posts placed in their posterior teeth (a total of 71 teeth in this case), and the second group was made up of the remaining 64 patients, all with 93 teeth in the anterior region. The follow-up was done twice at a six-month interval after cementation was complete.

Results: No significant changes were observed between the stability of the post structures in the anterior and posterior teeth that were examined, and the survival rates were found to be similar in both instances.

Conclusion: The cast metal posts performed at a similar success rate for the one-year period when the teeth were under our observation, and there were no considerable changes seen statistically. But it must be mentioned that a short follow-up period was observed, so the results might probably see some variations when a longer period of time is taken into consideration.

## Introduction

The placement of a post-and-core build-up is the standard of therapy for situations in which a tooth has undergone endodontic treatment and has subsequently lost both its structure and its vitality. The feel, experience, and empiricism of the practitioner have long been factors that influence the criteria used in the process of recovering teeth that have been endodontically treated. One of the things that must be taken into consideration before initiating endodontic therapy is an assessment of the tooth's capacity to be restored to its normal form and function. If restoration is selected, the patient's dentist has a number of post and core design options from which to choose in order to preserve the crown.

Posts have been used in nonvital teeth for over three centuries. The idea behind supports or dowels was to keep the coronal restoration in place if there was still enough tooth structure left. Posts were thought to be a way to strengthen non-vital teeth. According to several studies, the rigidity of the post should be as close to that of the root as possible in order to transfer occlusal stresses equally down the length of the root. Various designs and materials for posts and cores have been developed. Cast posts and cores, metal prefabricated posts with a composite resin core, and fibre posts with a composite resin core are all examples. Posts have been made from high noble metals, base metal alloys, zircon and quartz fibre, carbon fibre, and glass fibre composites. When there is extensive tooth structure loss or destruction, or irreversible pulpitis, root canal therapy (RCT) is recommended. The therapy is used to either remove the infection source or provide a way to reconstruct the coronal component of the tooth, or both [1-2]. A post may be required, depending on the amount of remaining coronal tooth structure, to allow the tooth crown to withstand intraoral forces during normal function [3]. The extent of hard-tissue destruction and the number of remaining axial cavity walls play a critical role in deciding whether to restore a tooth with a post prior to definitive restoration [4]. However, lab and research findings have found no evidence that a post can strengthen or prolong the life of endodontically treated teeth. The main objective of post-placement is to keep the core material in place.

Clinicians disagree on whether to use a custom-cast post-and-core or a prefabricated post. A gold alloy, silver-palladium alloy, or base metal alloy such as nickel-chromium alloy can be used to make a cast post-and-core (Ni-Cr). In teeth that cannot accommodate additional root preparation due to inadequate root dimensions or unsuitable internal morphology, such as mandibular incisors, a cast post-and-core is not recommended. A cast post-and-core is the best option when the angle of the core needs to be changed in relation to the root [5]. Regardless of the fact that cast post-and-cores have a very long and successful record, clinicians are increasingly using prefabricated posts due to their ease of use, reduced chair time, lower cost, and improved aesthetics [6]. Prefabricated posts are now available in a variety of materials, ranging from metallic (stainless steel and titanium alloys) to non-metallic (aluminium and plastic; zirconia and fibre-reinforced composite resin).

When there has been a significant loss of coronary dentin, a somewhat difficult method called post-and-core restorations is used to restore endodontically treated teeth in order to support retention and stability for the final restoration. According to Subash et al., the use of resin-modified glass ionomers as a core build-up material should be limited to non-stress-bearing areas. Because of its high fracture resistance and bonding to the tooth structure, composite resin is the preferred core build-up material for direct posts [7].

Over time, the materials used to cement posts into the canal have evolved. Zinc phosphate or an adhesive resin system can be used to cement metal posts. Adhesive cementation is preferred over zinc phosphate because it results in less microleakage, increased retention, and greater fracture resistance [8]. In a cohort of patients, Naumann et al. [4] showed that adhesively luted glass fibre and titanium posts used to restore endodontically treated teeth had a mean survival of up to 100 months, regardless of post material and rigidity.

In determining an appropriate treatment plan, the amount of remaining ferrule and tooth structure is critical. The amount of remaining coronal tooth structure and the tooth's ability to resist occlusal forces have been shown to be linked [9]. The ability to resist occlusal forces decreases as more tooth structure is removed, and the risk of fracture increases. The addition of a post frequently results in further tooth structure loss and a reduced ability to resist masticatory forces. This is complicated by the fact that the mechanical properties of the post-material influence post-and-core restorations (such as Young modulus). As a result, the post's material and design can affect the pattern of stress distribution throughout the tooth. Coelho et al. [10] concluded that stress was distributed differently between metallic and non-metallic posts based on a study using a finite element analysis (FEA) of weakened roots restored with various post materials. Endodontically treated teeth with glass and carbon fibre posts had a more homogeneous stress distribution, similar to that of an intact tooth, resulting in a lower risk of biomechanical failure. A Young modulus that is closer to the dentin is thought to result in better stress distribution and is often recommended. Because of the different force directions, biomechanical considerations suggest that anterior teeth, premolars, and molars will behave differently. Because of the greater horizontal forces, the maxillary region is considered a high-risk area for technical failures [11]. As a result, not only the component choice but also the tooth's location must be taken into account when making a choice. Post length, diameter, and design have all been reported to influence post-survival.

Various studies have favoured one post type over another, while others have found no difference between post types [12-13]. The authors are unaware of any consensus on which post type provides a higher rate of endodontically treated tooth survival. Systematic reviews comparing various post materials have produced contradictory findings. A meta-analysis of in vitro and in vivo studies found no significant differences between cast and direct post-and-core systems that would justify recommending one over the other [13]. Hence, it is debatable if tooth type, position in the dental arch, and choice of the post-and-core system affect the restoration of teeth that have had endodontic treatment.

This clinical study compared the clinical survival rates of cast metal post-and-cores made to order for anterior and posterior teeth while accounting for the population's age and gender, the type of post material, the length of the post, the amount of alveolar bone tissue supporting the roots, the location of the tooth in the dental arch, the type of cement used, the impact of the opposing dentition, and the type of definitive prosthetic treatment received. This study sought to determine whether the type of teeth receiving the restoration affected the post-and-core-survival rates in any way.

## Materials and methods

From April 2021 to April 2022, a prospective, parallel-group randomised clinical trial was conducted at Sadar Hospital with ethical committee clearance from Sadar Hospital and IRB number IEC/IRB/FDS/2022/04/07. At 90% statistical power, the sample size was determined using 0.5 standard deviations, a minimum anticipated difference of 0.52, and 0.05 significance. A total of 164 teeth were to be treated by 112 patients who had chosen post and core treatment following their RCT, regardless of their socioeconomic situation, religion, age, or sex. All of the posts used were cast metal, and the patients were separated into two groups: 48 patients had the posts inserted in their posterior teeth (a total of 71 teeth in this case), and the remaining 64 patients had the posts placed in their anterior teeth (a total of 93 teeth in this case). After the cementation was completed, the follow-up was done twice at a six-month interval. All therapy alternatives were thoroughly discussed with the patients, and their written agreement was obtained. Inclusion and exclusion have been explained in Table 1.

**Table 1:** Inclusion and exclusion criteria

Inclusion criteria
1. Intraradicular retention indicated for one or more endodontically treated anterior or posterior teeth.
2. Teeth with fully formed/fused roots.
3. Absence of any form of periapical/sinus pathology.
Exclusion criteria
1. History of untreated periodontitis, periodontal abscess or any other periodontal anomaly (or cases where pocket depth >2-3 mm)
2. Patients with serious medical issues for whom standard therapy and follow-up are not possible.
3. Patients who have known allergies to the products that will be utilised in the trial.

Table 2 shows the statistics of the number of teeth examined on the basis of their location and type.

**Table 2:** Number of teeth examined on the basis of its location and type used in the study

S.no	Tooth type	Number of teeth	Location of tooth	Baseline	After 6 months	After 1 year	Percentage of each category of teeth
1	Maxillary central incisor	18	Anterior	100%	98%	95%	10.97
2	Mandibular central incisor	9	Posterior	100%	98%	93%	5.48
3	Maxillary lateral incisor	22	Anterior	100%	97%	94%	13.41
4	Mandibular lateral incisor	13	Posterior	100%	98%	93%	7.92
5	Maxillary canine	18	Anterior	100%	97%	92%	10.97
6	Mandibular canine	13	Posterior	100%	98%	94%	7.92
7	Maxillary 1^st^ premolar	17	Anterior	100%	97%	92%	10.36
8	Mandibular 1^st^ premolar	13	Posterior	100%	96%	92%	7.92
9	Maxillary 2^nd^ premolar	5	Anterior	100%	97%	92%	3.04
10	Mandibular 2^nd^ premolar	7	Posterior	100%	95%	91%	4.26
11	Maxillary 1^st^ molar	9	Anterior	100%	96%	92%	5.48
12	Mandibular 1^st^ molar	11	Posterior	100%	96%	93%	6.70
13	Maxillary 2^nd^ molar	3	Anterior	100%	94%	92%	1.82
14	Mandibular 2^nd^ molar	6	Posterior	100%	95%	91%	3.65

**Figure 1:**
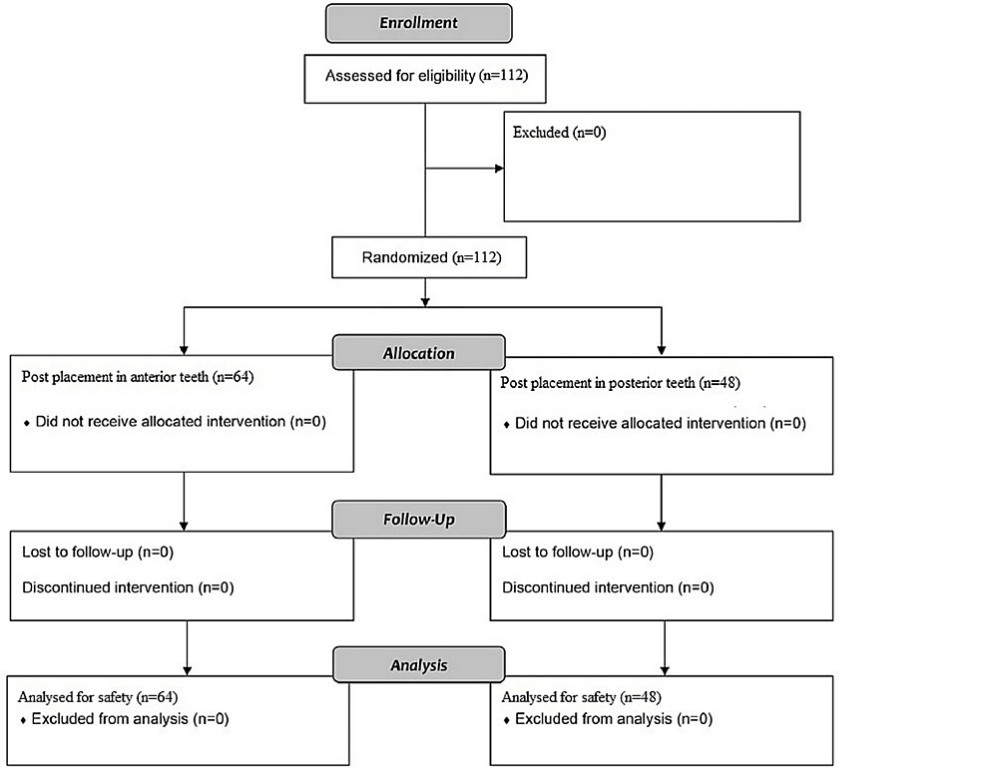
Flowchart representing randomisation and subject allocation for our investigation

Figure 1 represents the selection criteria of patients for this controlled trial and the subject allocation.

The SPSS statistical programme 26.0 version was used to analyze the data for this investigation (IBM, Inc., Chicago, USA). The mean and standard deviation were provided in the descriptive statistics. For the purposes of this investigation, the level of significance was set at 5%. The unpaired/independent t-test was used to provide an intergroup comparison for the difference in mean scores between two independent groups.

The subject allocation, results, discussion, and inferences hereby obtained from this investigation are in accordance with the CONSORT guidelines [14]. The CONSORT checklist was chosen because empirical data suggests that withholding the information is linked to skewed estimates of treatment impact or because the information is necessary to assess the validity or applicability of the findings.

## Results

Table 3 represents the statistical analysis of the outcomes depicted in the comparative study.

**Table 3:** Statistical analysis results of the findings obtained in this study (assuming the initial baseline success rate of 100%)

Follow-up period	Coefficients	Standard error	t Stat	P-value	Lower 95%	Upper 95%	Lower 95.0%	Upper 95.0%
After 1 year	0.43	0.16	2.65	0.02	0.07	0.80	0.07	0.80
After 6 months	0.50	0.17	2.94	0.01	0.12	0.88	0.12	0.88

Table 4 is a representation of the success rate of the post-and-core procedure of the anterior teeth used in this study on the basis of a six-month and one-year follow-up period.

**Table 4:** Success rate of anterior teeth after a six-month and one-year follow-up period (on the basis of a baseline success rate of 100% right after the post and core procedure)

Tooth type	Baseline	After 6 months	After 1 year	p-value
Maxillary central incisor	100%	98%	95%	0.02
Mandibular central incisor	100%	98%	93%	0.02
Maxillary lateral incisor	100%	97%	94%	0.02
Mandibular lateral incisor	100%	98%	93%	0.02
Maxillary canine	100%	97%	92%	0.02
Mandibular canine	100%	98%	94%	0.01

The success rate of posterior teeth in this study after post-implantation is depicted graphically in Table 5 after a six-month and one-year follow-up period.

**Table 5:** Success rate of posterior teeth after a six-month and one-year follow-up period (on the basis of a baseline success rate of 100% right after the post and core procedure)

Tooth type	Baseline	After 6 months	After 1 year	p-value
Maxillary 1^st^ premolar	100%	97%	92%	0.01
Mandibular 1^st^ premolar	100%	96%	92%	0.01`
Maxillary 2^nd^ premolar	100%	97%	92%	0.01
Mandibular 2^nd^ premolar	100%	95%	91%	0.01
Maxillary 1^st^ molar	100%	96%	92%	0.01
Mandibular 1^st^ molar	100%	96%	93%	0.01
Maxillary 2^nd^ molar	100%	94%	92%	0.01
Mandibular 2^nd^ molar	100%	95%	91%	0.01

There were no periodontal complications with any of the teeth, and no endodontic-related pain was recorded. One failure in the cast metal group was found to be caused by debonding at the post-cement junction.

## Discussion

Our findings support those of a randomised controlled experiment that evaluated the survival rates of anterior and posterior teeth treated with cast metal post-and-cores without any residual coronal wall and showed that post type did not significantly affect the survival of restorations [15]. In this investigation, the post type had no bearing on the survival rates of post and core repairs. Cloet et al. [16], on the other hand, demonstrated that front teeth fail more frequently than posterior teeth. Other investigations that also noted an increase in the frequency of anterior tooth fractures have confirmed this finding [17-18]. Occlusal forces are typically responsible for these fractures. Also, our investigation did not find any evidence of the 3 times increased fracture risk of anterior teeth treated with post-and-core restorations compared to posterior teeth as reported by a particular study, although it is likely due to the use of a different material as compared to the cast metal post and core used in our study [19].

Additionally, another study [20] found that tooth position had no impact on survival rates. These findings conflict with those of our study, which found that neither the anterior nor the posterior teeth had a higher frequency of failure. Instead, our results concur with those of investigations by Torbjorner et al. [21]. The angulation of the teeth, which leaves them vulnerable to shear stresses and eccentric loading, is one explanation for why anterior teeth fail more frequently, as observed in investigations that did not concur with the results that we obtained. To improve the survival rates of teeth with post-and-core restorations, which were used in our study, the significance of a ferrule and the preservation of coronal tooth structure have been emphasised [22-23].

Traditional root canal fillings, whether with or without a post and core, provide little or no support for the rebuilt tooth. One-millimetre ferruled teeth restored using a cast post-and-core system, a Composipost post and composite resin core system, or a stainless steel post and composite resin core system demonstrated better resistance to fracture than nonvital endodontically treated teeth [24].

A few limitations are linked to this study because of the large number of variables. Because this was a retrospective study, the post-preparation and cementation protocol, as well as the materials utilised, were not standardised, and the same clinician was not used. The treatment protocol most likely differed from provider to provider, which could affect the survival rate. The post material was not always mentioned in the chart, so it had to be calculated based on the radiographs. The radiographs examined were a mix of traditional film radiographs and digital radiographs scanned into the computerised chart. All radiographs were taken using a film holder and a beam alignment device, as per the usual procedure. Although radiographs show that prefabricated metal and fibre-reinforced composite resin posts have similar looks, some posts may have been misinterpreted. Furthermore, some of the criteria analysed are difficult to measure (for example, the proportion of root surrounded by bone), introducing some investigator bias into the parameters' subjectivity. Because post space width was not documented during preparation, it was not possible to compare posts based on narrow versus flared anatomy. There was no mention of hygiene, caries risk, or bone loss over time. For example, if a patient's caries risk is higher than another's, secondary caries failure is more likely than in a patient with a low caries risk. Furthermore, the amount of ferrule remaining and the number of dentinal walls preserved were not documented, both of which are indicators of restorative failure.

## Conclusions

Metal posts are used the majority of the time in a mutilated tooth and hence play an important role in saving the tooth. During the course of the one year that the teeth were under our scrutiny, during which time we monitored their performance, there were no statistically significant differences seen in the performance of the cast metal posts. Although it is important to point out that just a small amount of time was spent following up on the participants, the findings should still be taken with a grain of salt due to the fact that there is a good chance that they will change when looking at them over a longer period of time.
